# Restoring Sensation through Abdominal Flap Neurotization in Breast Reconstruction

**DOI:** 10.3390/jcm13133826

**Published:** 2024-06-29

**Authors:** Max L. Silverstein, Arash Momeni

**Affiliations:** Division of Plastic and Reconstructive Surgery, Stanford University School of Medicine, Palo Alto, CA 94304, USA

**Keywords:** neurotization, breast reconstruction, reinnervation

## Abstract

Breast sensation plays a significant role in the safety and quality of life of women who undergo mastectomy and reconstruction. In 1992, Slezak et al. introduced the concept of abdominal flap neurotization to improve sensation of the reconstructed breast. Over the next 30 years, numerous studies iterated on Slezak’s technique, suggesting technical modifications and new methodologies for assessing sensory recovery. Despite evidence that reinnervation increases patient satisfaction following autologous breast reconstruction, abdominal flap neurotization remains a rarely performed procedure. In this article, we review the evolution of flap neurotization in breast reconstruction and describe our approach to facilitating sensory recovery of the breast while limiting donor site morbidity.

## 1. Introduction

As microsurgical breast reconstruction has become a routine part of breast cancer care, the expectations of both surgeons and patients have exceeded the simple restoration of a soft tissue mound. Sensation of the reconstructed breast has been shown to play an important role in patient safety and satisfaction. Numerous reports have documented the risk of accidental injury secondary to insensate reconstructed breasts [1,2,3,4,5,6,7]; simultaneously, a growing body of patient-reported outcome (PRO) studies has conveyed the psychosocial value of breast sensation to women who undergo mastectomy and autologous reconstruction [8,9,10,11]. Despite these benefits, routine neurotization of the reconstructed breast remains uncommon even in major breast cancer treatment centers, partly due to a lack of consensus on the optimal surgical technique or methodology for evaluating sensory outcomes [12]. In this article, we review the literature on restoring sensation in microsurgical breast reconstruction and describe our preferred technique for achieving reinnervation of the reconstructed breast.

## 2. Background

Ten years after Hartrampf described the transverse rectus abdominis myocutaneous (TRAM) flap for postmastectomy breast reconstruction [13], Slezak et al. introduced the concept of TRAM flap neurotization in their innovative 1992 article on restoring breast sensation [14]. In that study, the authors described isolating lower intercostal nerves during abdominal flap elevation and coapting the stumps to the lateral mammary rami of the fourth intercostal nerve at the chest. They reported earlier recovery of vibratory sensation in surgically reinnervated TRAM flaps and proposed that neurotization might offer significant benefit over standard reconstruction. Five years passed before the next report on TRAM flap neurotization by Doncatto et al., who showed that 27 out of 27 neurotized TRAM flap patients demonstrated superficial sensation at 8 months compared to only 3 out of 27 non-neurotized patients [15]. In 1999, Blondeel et al. published the first description of free abdominal flap neurotization and argued that nerve coaptation increased the quality and quantity of sensation in autologous breast reconstruction [16].

Simultaneously, a number of studies reported on spontaneous recovery of sensation in breasts reconstructed using autologous tissue, implying that surgical breast neurotization might not be necessary [17,18,19,20,21,22,23,24]. Those studies, however, were characterized by inconsistent methodologies and imprecise definitions for the satisfactory recovery of breast sensation. Moreover, their findings indicated that while most non-neurotized free flaps regained some sensation within 12 months, others remained insensate years after surgery with a wide variation in the timing and degree of recovery [22,23,24,25,26,27]. Concurrently, a series of articles documented thermal injuries associated with numbness of breasts reconstructed with autologous tissue, demonstrating the safety benefit of breast sensation [1,2,3,4,5,6,7]. Since the early 2000s, the literature has become increasingly resolute, with numerous primary studies and systematic reviews concluding that neurotized flaps for breast reconstruction display earlier, more consistent, and more complete sensory recovery, measured through a variety of testing methodologies [8,9,14,15,16,19,20,21,22,23,24,25,26,27,28,29,30,31,32,33,34,35,36,37,38,39,40].

Blondeel’s 1999 article was also the first to report patient satisfaction scores among women undergoing neurotized versus non-neurotized abdominal flap breast reconstruction [8]. They found that patients whose flaps were neurotized were more likely to report subjective return of sensation, and especially erogenous sensation, compared to non-neurotized counterparts at 20-month follow-up. They speculated that while sensation to pressure and touch might return through ingrowth of nerve fibers from wound edges alone, the recovery of erogenous sensation seemed dependent on surgical nerve coaptation. In the years that followed, a number of PRO studies expanded on these findings, consistently corroborating the conclusion that neurotization improves patient satisfaction after breast reconstruction [9,10,11,27].

## 3. Relevant Anatomy

The breast derives sensation from the anterior cutaneous branches of the second to sixth intercostal nerves (ICNs), the lateral cutaneous branches of ICNs three through six, and supraclavicular branches of the cervical plexus [14,41,42,43]. The ICNs travel along the inferior borders of the ribs, splitting into lateral cutaneous branches that give off anterior rami to innervate the lateral trunk, and continuing medially towards the sternum where they become anterior cutaneous branches that supply the medial chest. While ICNs contain a mixture of sensory and motor fibers in their main trunks, the lateral and anterior cutaneous branches are almost exclusively sensory [44]. A recent study demonstrated similar axonal counts between the lateral and anterior cutaneous branches on histological analysis of cadaveric specimens [43].

The nipple-areolar complex (NAC) is most consistently innervated by the lateral cutaneous branch of the fourth ICN, with more minor contributions from the third and fourth anterior cutaneous branches [42,45]. While the lateral cutaneous branch is often disrupted during mastectomy as it travels through the breast parenchyma towards the NAC, the anterior cutaneous branch is typically preserved, superficializing near the sternum and then traveling toward the nipple within the pre-mastectomy-plane subcutaneous tissues [14,42,45,46]. ICN 3 can be identified running along the lower border of the third rib near the sternum, crossing superficially over the internal mammary vessels before giving off its anterior cutaneous branch [43,47].

The abdominal wall receives sensory and motor innervation from the thoracolumbar ICNs, with the infraumbilical segment supplied by T10 through L1 [48]. The ICNs travel ventrally in a plane between the transversus abdominis and internal oblique muscles before entering the rectus sheath at its lateral margins. The nerves then join a longitudinal plexus running craniocaudally with a lateral branch of the deep inferior epigastric artery (DIEA) along the deep surface of the rectus abdominis muscle [48]. The nerves penetrate the muscle at a variable location relative to the abdominal midline, coincident with the lateral-most row of the DIEA [48]. Within the rectus abdominis muscle, the ICNs split into motor branches, which power the muscle, and sensory branches, which join perforating vessels as they course toward the skin [42,46,49]. Sensory branches can be identified traveling with perforators from both the medial and lateral rows of the DIEA [48,50].

Rozen and colleagues are responsible for much of our anatomical understanding of the abdominal wall in the context of free flap harvest [48,49,51]. In 2008, they challenged the classical notion that segmental branches of the ICNs directly innervate corresponding transverse sections of the rectus abdominis muscle. Instead, their anatomical study using nerve stimulation revealed the presence of two distinct categories of motor nerves within the rectus: type 1 nerves, which supply narrow longitudinal strips of muscle and have abundant overlapping innervation from nearby branches, and type 2 nerves, which are larger, usually found near the arcuate line, and innervate entire transverse strips of the muscle, without significant redundancy [49]. They concluded that while some (type 1) ICN motor branches could be sacrificed without functional detriment, other (type 2) nerves were critical to maintaining abdominal wall integrity and should be preserved whenever possible [48,49].

## 4. Surgical Techniques

In Slezak et al.’s original article on TRAM flap neurotization, the authors described isolating a 4 to 6 cm segment of the abdominal ICN bundle during flap harvest, which could be directly coapted to the anterior ramus of the lateral cutaneous branch of the fourth ICN at the chest [14]. This technique was replicated, largely unchanged, in the majority of subsequent studies on TRAM flap reinnervation. The lateral cutaneous branch of ICN 4 continues to be the preferred recipient target of many surgeons due to its role as the primary sensory nerve to the NAC [42,45,52]. Its location, however, at the lateral chest wall, necessitates a second point of flap attachment distant from the usual vascular microanastomoses to the internal mammary vessels, severely restricting flap mobility. Additionally, direct coaptation to the lateral cutaneous branch requires a long segment of donor nerve, which can be obtained only by harvesting the abdominal ICN at a point proximal to its motor–sensory bifurcation [48,49,53]. Finally, the lateral cutaneous branch runs through the breast parenchyma and preservation of a viable recipient stump is dependent on the technique of the oncologic surgeon [28,43].

In response to these issues, Spiegel et al. proposed DIEP flap neurotization via coaptation to the anterior branch of the third ICN near the sternum [38,54,55]. In their 2009 and 2013 articles, the authors described dissecting through pectoralis major to isolate the third ICN as it runs along the third rib and over the internal mammary vessels. The nerve was divided and coapted to the donor ICN, either directly end-to-end or with the use of a nerve conduit [38]. Use of the anterior cutaneous branch as the recipient nerve confined all microsurgical connections to a single field per side, improving surgical efficiency and facilitating flap mobility on inset [38,47,53,54,55,56].

The harvest of long segments of ICN during flap elevation seemed for many years to be an unfortunate inevitability of abdominal free flap neurotization. Despite only utilizing the sensory component of the donor ICN, the need for adequate length forced surgeons to isolate the nerve at a point proximal to its bifurcation into motor and sensory branches within the rectus muscle. In so doing, reinnervation of the breast threatened to result in denervation of the abdominal wall, increasing the potential for complications like abdominal bulge or hernia [49,57]. To address this problem, in 2018, the senior author (A.M.) introduced a novel method for harvesting only a sensory branch to the abdominal flap tissue, thereby preserving motor innervation of the abdominal wall [47].

**Figure 1:**
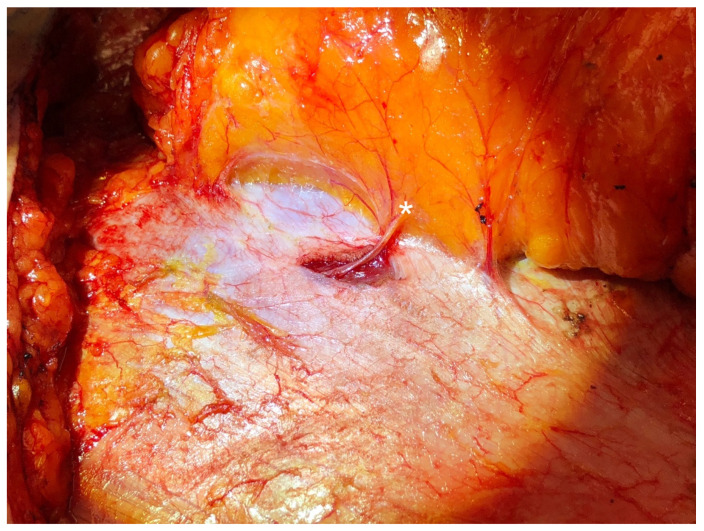
A sensory intercostal nerve branch (denoted by the asterisk) that is not traveling with the dominant perforating vessels is harvested via a short separate fascial incision during abdominal flap elevation.

In this technique, sensory nerves are identified traveling with lateral row perforating vessels toward the skin during standard abdominal flap elevation. If the flap is to be based on lateral row vessels, the anterior rectus sheath is incised in line with these perforators, as is commonly performed in DIEP flap harvest. Retrograde dissection of a centrally located sensory nerve is performed until a sensory–motor Y-junction is encountered, typically representing a bifurcation of ICN 11 or 12. The sensory branch is clipped and divided distal to the Y-junction, thus, preserving the motor branch to the rectus abdominis muscle. If the flap is based on medial perforators, a lateral sensory nerve branch is isolated and harvested via a separate 5 mm incision in the fascia (Figure 1), which is closed with a single figure-of-eight stitch [47,53,56]. The rest of flap elevation and pedicle dissection proceed in standard fashion.

**Figure 2:**
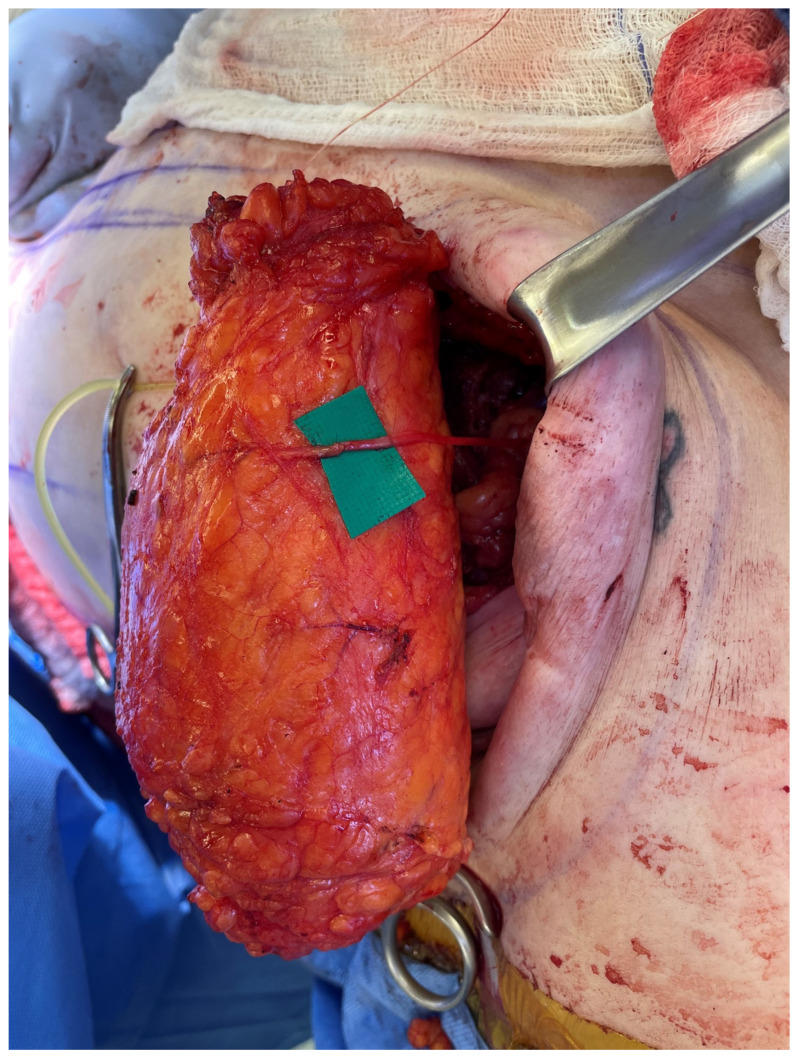
The abdominal sensory branch harvested with the flap is often short (<3 cm) and, therefore, coapted to an interpositional nerve allograft to facilitate tension-free repair.

**Figure 3:**
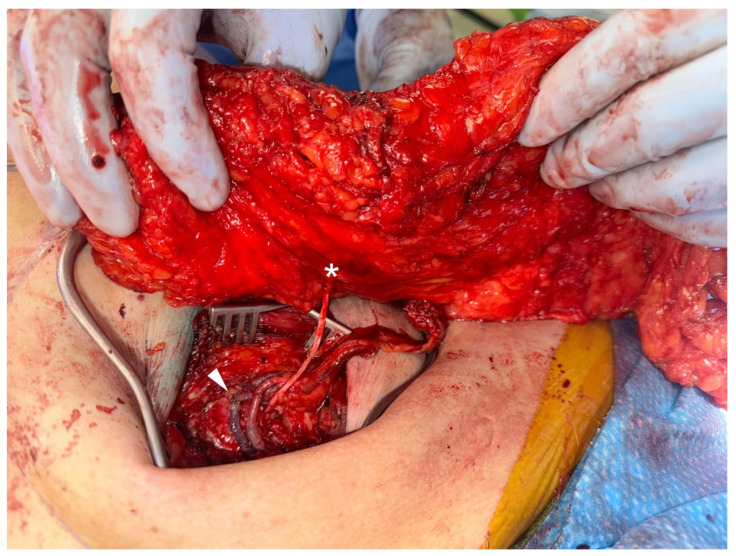
The flap sensory branch (denoted by asterisk) is coapted via bridging nerve allograft to the anterior cutaneous branch of intercostal nerve 3 near the sternum, adjacent to the microvascular anastomoses (denoted by the arrowhead).

The sensory-only nerve branch harvested using this technique is typically no longer than 3 cm and, therefore, too short to reach recipient targets; to address this issue, our group has described a second innovation: the use of bridging nerve allograft to facilitate tension-free coaptation to the anterior cutaneous branch of ICN 3 in the chest (Figure 2) [47,53,56]. Following mastectomy, the pectoralis major is split over the third rib, and the third costal cartilage is removed in typical fashion. The posterior perichondrium is incised and reflected medially, facilitating exposure of ICN 3 running along the inferior border of the rib as it courses superficially over the internal mammary vessels. Of note, ICN 3 can also be used if a rib-sparing approach is utilized. The nerve is divided at the level of the internal mammary artery and reflected laterally for coaptation. A 1–2 mm × 50 mm segment of processed human nerve acellular allograft (Avance, AxoGen, Alachua, FL, USA) is coapted proximally end-to-end to the anterior cutaneous branch of ICN 3. Next, the abdominal flap is transferred to the chest and microvascular anastomoses to the internal mammary vessels are performed in standard fashion. Following flap revascularization, distal nerve coaptation to the sensory branch of ICN 11 or 12 supplying the flap is performed (Figure 3). Epineurial repair is performed using 9-0 nylon suture without nerve wraps. Importantly, this sequence—performing the proximal nerve coaptation prior to flap transfer and distal nerve coaptation after flap revascularization—minimizes flap ischemia time. The flap is then inset after the excision of all flap skin, including both the epidermis and dermis [47,53,56].

In addition to sparing important motor innervation to the abdominal wall, this technique ensures that only an afferent donor branch is involved in the coaptation, focusing reinnervation on the regeneration of sensory fibers. Nerve allograft is an ideal material for bridging the 35–50 mm gaps typically encountered in breast flap neurotization using our technique. An abundance of literature supports the notion that nerve allograft is noninferior to autograft for bridging gaps of up to 70 mm in peripheral nerve repair [58,59,60]. While nerve autograft is considered the gold standard for bridging longer gaps, graft harvest can result in significant donor site morbidity, including the potential for neuroma formation [61,62,63]. Moreover, the costs associated with nerve allograft and nerve autograft are similar after accounting for the additional operating room time and resources required to harvest donor nerves [61,64].

Spiegel et al. reported success using 40 mm hollow tube nerve conduits during DIEP flap neurotization; most peripheral nerve research, however, recommends the use of nerve conduits only for gaps of less than 10 mm, and Spiegel’s technique has not been replicated [38,60,65]. Djohan et al. recently published encouraging results after performing neurotized breast reconstruction with a bridging nerve allograft to the third or fourth anterior ICN in combination with nerve conduits, though the specific benefit of the conduits is unclear [66]. In our experience, coaptation of the sensory branch of ICN 11 or 12 to the anterior cutaneous branch of ICN 3 via an interpositional nerve allograft dependably results in a greater degree of sensory recovery to the reconstructed breast relative to non-neurotized controls [53].

In our hands, abdominal flap neurotization using interpositional nerve allograft adds less than 15 min of surgical time per side. Other studies report that neurotization adds 10 to 31 min to the duration of autologous breast reconstruction [10,29,35,37,55]. Xia et al. recently compared neurotized versus non-neurotized abdominal flap breast reconstructions over 73 patients and found a non-statistically significant difference in operative time (467.73 ± 145.52 versus 455.28 ± 111.19 min) [67].

Table 1 conveys the most important technical advances that have occurred during the short history of neurotization in abdominal free flap breast reconstruction.

## 5. Outcomes

Breast reinnervation research is characterized by heterogeneity in terms of how sensory recovery is measured and which types of sensation (e.g., protective, erogenous, vibratory, etc.) are evaluated. As a result, systematic reviews on the topic are often solely descriptive and unable to perform meaningful meta-analysis on a comprehensive collection of articles [10,47,68,69]. Despite the variability, studies are overwhelmingly supportive of the beneficial effects of surgical breast flap neurotization, both in terms of objective testing and PROs.

Slezak’s original article measured breast sensation by using Semmes–Weinstein monofilaments (SWMFs) to evaluate pressure sensitivity and a biothesiometer to determine the vibratory detection threshold at nine defined points corresponding to the quadrants of the breast and NAC [14]. While SWMF testing has been criticized by some authors for its logarithmic (rather than linear) progression and potential for inter-observer inconsistency [54,70], it offers the benefit of being widely available, non-invasive, and easy to perform with minimal training. SWMF testing is also inherently meaningful, with established values corresponding to the presence of protective sensation, though these thresholds are derived from research on the hand, not the breast [70,71]. Numerous studies have used SWMF testing to demonstrate clinically meaningful recovery of pressure sensitivity in neurotized breasts that occurs earlier (usually within 12 months), more consistently, and to a greater degree relative to non-neurotized controls [9,11,14,16,21,22,23,24,26,27,28,29,30,31,32,33,35,37,53].

Several authors have evaluated breast sensory recovery using the Pressure-Specifying Sensory Device (PSSD), an instrument developed by Dellon in the early 1990s that consists of two prongs mounted on force transducers which measure the precise pressure required to elicit perception, as calculated by a computer [72]. The PSSD is highly sensitive and specific in its typical applications for diagnosing nerve compression syndromes and is more consistent compared to SWMF testing [72,73]. Its proponents also point to the PSSD’s ability to determine exact pressure sensitivity thresholds for both moving and static stimuli, where SWMFs provide only an estimated lowest perceivable value for static forces [70]. Several studies have utilized the PSSD to evaluate reinnervation of the reconstructed breast with results that largely echo those derived from SWMF testing [25,34,38,66]. While the PSSD is clearly beneficial for detecting small sensory changes in cases of upper extremity nerve compression, its superiority over slightly less precise devices in the context of breast reinnervation is less obvious. In our opinion, the SWMF’s ubiquity and ease of use make it an indispensable tool for evaluating breast neurotization outcomes.

Other studies have tested breast reinnervation by measuring 2-point discrimination [9,14,23,25,28,29,33,34,35,37], vibration sensitivity [8,14,33,37], and sharp/blunt differentiation [28,29,33,37]. Given the well-documented incidence of thermal injuries to insensate reconstructed breasts, several researchers have tried to evaluate the effect of neurotization on temperature sensitivity, with mixed results. While Temple et al. found that patients with neurotized TRAM flaps were better able to discriminate between test tubes filled with either 43 °C or 16 °C water at 15-month follow-up [35], most other studies have not identified a significant difference in the temperature sensitivity of neurotized versus non-neurotized flaps [15,26,27,30,33,37]. Heterogeneity in the measurement methodology used across various studies obscures definitive conclusions about some of the more nuanced aspects of sensory recovery, including the ability to detect contact with dangerously hot substances.

Most importantly, perhaps, PRO studies have consistently demonstrated quality of life benefits associated with breast neurotization. Blondeel et al. published the first PRO study on the topic in 1999, showing that women who underwent neurotized DIEP flap reconstruction were more likely to report recovery of breast sensation, including erogenous sensation, compared to women with non-neurotized flaps at 20-month follow-up. In 2009, Temple et al. authored a seminal paper in which they used three validated, quality of life questionnaires to assess the psychosocial effects of neurotization in free TRAM flap breast reconstruction [9]. At 4 years post-reconstruction, they found that women who received neurotized flaps reported significantly greater satisfaction in terms of physical function, physical role, general health, social function, and emotional role, compared to non-neurotized counterparts. They also found that patients with innervated flaps reported significantly better quality of life on the Functional Assessment of Cancer Therapy—Breast instrument. Then, in 2018, Cornelissen and colleagues evaluated DIEP flap neurotization using the BREAST-Q module and found that flap reinnervation was associated with an 18 percent higher average score in the ‘Physical Well-Being of the Chest’ domain [11]. Despite variability in the instruments used to evaluate patient satisfaction following neurotized breast reconstruction, outcomes are consistent: surgical reinnervation improves patient quality of life following autologous breast reconstruction.

## 6. Future Directions

Reinnervation in autologous breast reconstruction remains an incompletely understood topic with ample room for further research and technical improvement. While this article focuses on abdominal flap breast reconstruction, alternative flaps have also been neurotized, including the latissimus dorsi [74], profunda artery perforator [75], and superior gluteal artery perforator flaps [69]. Further studies are needed to better understand the relative potential for reinnervation of these flaps and any techniques for optimizing sensory recovery. Similarly, little is known about improving the sensation of breasts following alloplastic reconstruction, though it is an area of active investigation.

Further research should also aim to clarify the processes underlying the phenomenon of breast reinnervation. While early studies measured sensation on abdominal flap skin paddles following skin-sparing mastectomy (SSM), more recent studies have tested for sensory recovery of the native breast skin after nipple-sparing mastectomy (NSM) followed by reconstruction using de-epithelialized abdominal tissue. This transition mirrors the overall trend toward NSM in the United States [76] but raises important questions about the comparability of historical data to more contemporary studies and the effect of mastectomy type on outcomes [70]. Moreover, little is known about the exact mechanism by which abdominal flap neurotization improves sensation in native breast skin, or whether mastectomy skin thickness may affect the potential for reinnervation [70]. The theory that regenerating nerves within the abdominal tissue have a direct effect on the overlying mastectomy flap informs our practice of removing both epidermis and dermis from neurotized abdominal flaps in an effort to facilitate contact with the native breast skin [47,53]. Better characterization of the process by which breast skin reinnervation occurs, especially in the setting of neurotized free flap reconstruction, should inform improvements in surgical techniques and enhance patient outcomes.

Despite the currently available evidence that neurotization improves outcomes of autologous breast reconstruction, neurorrhaphy is performed in less than 2 percent of autologous breast reconstruction procedures [12]. The explanation for this lack of adoption is almost certainly multi-factorial, involving a perceived lack of benefit, concerns about the operative time required, and fear of associated morbidity. Abdominal flap neurotization is a safe procedure, with virtually zero reports of symptomatic neuroma or flap compromise as a result of nerve coaptation [39,54]. A large database study performed by Laikhter et al. found no association between neurotization and any complication, return to the operating room, or prolonged hospital stay following autologous breast reconstruction [12]. As previously discussed, neurotization has a relatively minor impact on the overall operative duration of autologous breast reconstruction, especially considering the well-established benefits of breast reinnervation. To facilitate increased adoption of neurotization, futures studies should present data from randomized trials with practical surgical techniques and standardized methods of evaluating sensory and patient-reported outcomes.

## 7. Conclusions

The currently available evidence supports the notion that breast sensation plays a significant role in the safety and quality of life of women who undergo mastectomy and reconstruction. Since Slezak introduced the concept of TRAM flap neurotization three decades ago [14], iterative advances have improved the efficiency and effectiveness of breast reinnervation surgery and the methods used for assessing outcomes. Despite this progress, less than 2 percent of autologous breast reconstruction procedures performed today involve neurotization [12]. Increased adoption depends on continued refinement of neurotization techniques and standardization of protocols for assessing reinnervation. Restoring breast sensation after mastectomy represents an opportunity for plastic surgeons to dramatically improve one of our specialty’s core operations.

## Figures and Tables

**Table 1:** TRAM, transverse rectus abdominis myocutaneous; ICN, intercostal nerve; LCB, lateral cutaneous branch; ACB, anterior cutaneous branch.

Authors	Year	Technical Innovation	Advantages
**Slezak et al. [14]**	1992	Introduced the concept of TRAM flap neurotization (via coaptation of a long segment of abdominal ICN to the LCB of ICN 4)	Accelerates the return of sensation in TRAM flaps versus those that do not undergo surgical neurotization
**Spiegel et al. [38,55]**	2009, 2013	Described utilizing the ACB (rather than the LCB) of ICN 3 as a recipient nerve	Confines all microsurgical connections to a single field, improving efficiency and flexibility of flap inset
		Used 40 mm hollow tube nerve conduits to bridge gaps between donor and recipient nerves	Enables nerve coaptation over the longer distances associated with using the ACB as a recipient; reportedly improves sensory recovery outcomes versus neurotization via direct coaptation
**Puonti et al. [33]**	2017	Performed dual neurorrhaphy from the donor ICN 10–12 to both the ACB of ICN 3–4 (medially) and either the thoracodorsal, intercostobrachial, or LCB of ICN 4–5 (laterally)	Enhances tactile, temperature, vibratory, and overall sensory scores relative to single neurorrhaphy TRAM flap neurotization
**Zhou et al. [47]**	2018	Described harvesting a short, sensory-only segment of ICN 11 or 12 with the abdominal flap	Preserves motor innervation to the abdominal wall and focuses reinnervation on afferent fibers
		Introduced the use of interpositional nerve allografts to bridge between short donor nerves and the ACB of ICN 3 in the chest	Enables neurorrhaphy over the longer distances that result from harvesting short sensory-only sections of abdominal ICN, without introducing donor site morbidity associated with nerve autograft harvest
**Momeni et al. [53]**	2021	First study investigating long-term outcomes following flap neurotization with interpositional nerve allografts	Flap neurotization using nerve allograft allowed for tension-free and selective nerve coaptation to sensory nerve fibers, which resulted in improved return of protective sensation
**Djohan et al. [66]**	2023	Performed flap neurotization using both an interpositional nerve allograft and nerve conduits to cover each coaptation site	Nerve conduits theoretically collect leaked neurotrophic factors at nerve ends while providing a protected environment for more effective nerve regeneration
